# Longitudinal mucosal incision prior to balloon dilation: Novel and advanced approach for severe esophageal stenosis

**DOI:** 10.1055/a-2569-8517

**Published:** 2025-05-12

**Authors:** Ippei Tanaka, Gantuya Boldbaatar, Kei Ushikubo, Kazuki Yamamoto, Yohei Nishikawa, Mayo Tanabe, Haruhiro Inoue

**Affiliations:** 1 378609 Digestive Diseases Center, Showa University Koto Toyosu Hospital, Koto, Japan

Stricture is a common complication following esophageal endoscopic submucosal dissection (ESD), occurring in 94.1% of cases involving resection of three-quarters or more of the circumference if no preventive measures are implemented. 1 2 . Standard treatments, such as balloon dilation with local or systemic steroid administration, are recommended in current guidelines 3 . However, some refractory strictures remain unresponsive, significantly reducing patient quality of life to such measures. This report presents a novel endoscopic approach to treat such cases.

A 70-year-old male underwent ESD for a circumferential esophageal cancer followed by 100 mg of triamcinolone injection at another hospital. However, strictures developed 1 month later ( Fig. 1 **a** ). Subsequent balloon dilations were performed biweekly without improvement, leading the patient to seek care at our hospital.

**Fig. 1:**
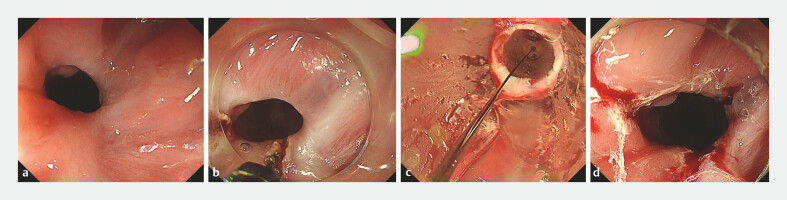
**a** Refractory esophageal stenosis can be seen, which prevents scope passage. **b** A longitudinal mucosal incision was carefully made on the stenosis site. **c** Subsequently, balloon dilation was performed to 15 mm. **d** There was no sign of perforation after the procedure.

Upon performing balloon dilation again for the stricture measured about 8 mm, we identified that same lacerations were consistently forming only in the 4 o’clock direction. Therefore, we adopted a strategy of performing mucosal incisions prior to balloon dilation to redistribute pressure applied to the stricture site during dilation ( Video 1 ). Endoscopic ultrasonography using a miniature probe (UM-S20–17R) was performed preoperatively to assess wall thickness.

This video shows a new method of longitudinal mucosal incision prior to balloon dilation for refractory esophageal stenosis caused by circumferential endoscopic submucosal dissection.Video 1

Using a needle knife (KD-645L: Olympus), longitudinal mucosal incisions were carefully made on the stricture at the 6 o’clock and 8 o’clock positions ( Fig. 1 **b** ). Balloon dilation then was performed using a balloon device (KD-645L, 12 to 15 mm: Olympus) to expand the stricture to 15 mm ( Fig. 1 **c** ). Lacerations at the incision points were extended and additional tearing was observed at the 4 o’clock position, which was less severe than in previous procedures as we expected ( Fig. 1 **d** ). Subsequently, 40 mg triamcinolone was injected into the incision site. Four weeks later, follow-up endoscopy revealed that the stricture had resolved, allowing the endoscope to pass through smoothly ( Fig. 2 ). This improvement was still evident at 8-week follow-up.

**Fig. 2:**
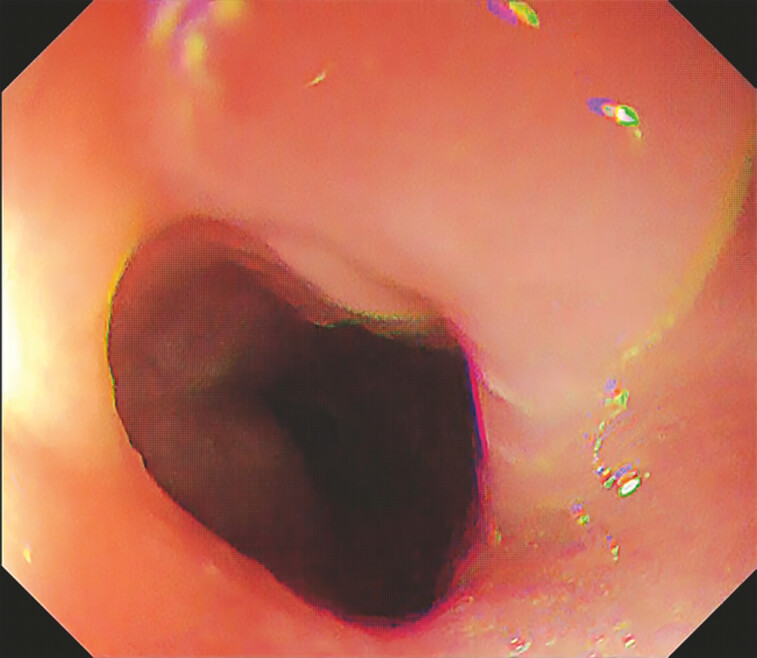
This image shows the stenosis site 1 month after the procedure. The endoscope could be passed through it.

Balloon dilation often fails to apply pressure in various directions at the stenosis site, which may be a major cause of lack of improvement. Longitudinal incisions at other points can distribute the pressure during dilation, which may offer promising new treatment for refractory stenosis cases.

## Notes

Correction **Longitudinal mucosal incision prior to balloon dilation: Novel and advanced approach for severe esophageal stenosis** Ippei Tanaka, Gantuya Boldbaatar, Kei Ushikubo et al. Endoscopy International Open 2025; 13: a25698517. DOI: 10.1055/a-2569-8517 In the above-mentioned article the author names Gantuya Boldbaatar and Mayo Tanabe were corrected. This was corrected in the online version on 21.05.2025.
