# Correction: Longitudinal mucosal incision prior to balloon dilation: Novel and advanced approach for severe esophageal stenosis

**DOI:** 10.1055/a-2616-2611

**Published:** 2025-05-21

**Authors:** Ippei Tanaka, Gantuya Boldbaatar, Kei Ushikubo, Kazuki Yamamoto, Yohei Nishikawa, Mayo Tanabe, Haruhiro Inoue

**Affiliations:** 1 378609 Digestive Diseases Center, Showa University Koto Toyosu Hospital, Koto, Japan



In the above-mentioned article the author names Gantuya Boldbaatar and Mayo Tanabe were corrected. This was corrected in the online version on 21.05.2025.

